# Hydromorphological evaluation of the river training impact on a multi-thread river system (Belá River, Carpathians, Slovakia)

**DOI:** 10.1038/s41598-021-85805-2

**Published:** 2021-03-18

**Authors:** Anna Kidová, Artur Radecki-Pawlik, Miloš Rusnák, Karol Plesiński

**Affiliations:** 1 Institute of Geography, Slovak Academy of Sciences, Štefánikova 49, 81473 Bratislava, Slovakia; 2 Faculty of Civil Engineering, Cracow University of Technology, ul. Warszawska 24, 31155 Krakow, Poland; 3 Faculty of Environmental Engineering and Land Surveying, University of Agriculture in Krakow, al. Mickiewicza 24/28, 30059 Krakow, Poland

**Keywords:** Geomorphology, Environmental impact, Hydrology

## Abstract

The paper evaluates the impact of river training works designed to address problems associated with flooding on the braided-wandering Belá River in Slovakian Carpathians. This impact was investigated after the flood event in July 2018 on 11 river reaches where the river engineering and management intervention was applied. We analyzed its impact by spatio-temporal variations in river morphology (12 channel parameters) and changes in cross-section and hydraulic parameters (flow velocity, shear stress, stream power, W/D ratio) between pre- and post-flood management periods. The research hypotheses related to decreasing geodiversity in managed river reaches, a rapid increase in flow velocity during an extreme flood in river reaches where there is no sufficient floodplain inundation due to artificially high banks built by river training works, and increasing erosive force in the channel zone thanks to river management intervention were confirmed. The intervention in the braidplain area of the Belá River resulted in an undesirable simplification of the river pattern, loss of geomorphic diversity, loss of channel–floodplain connectivity, and disturbance and restraint of hydromorphological continuity. Identification of main conflicts of the Belá River management is important for clarifying the different approaches of stakeholders in the study area and aims to provide an objective illustration of their consequences. The presented analyses could help in future management issues as well as in the more critical decision-making process in vulnerable and rare braided river systems on the present when we are losing so many natural rivers by human decisions.

## Introduction

The multi-thread pattern of a high-energy river^1^ reflects a natural bedload pulse system^2^ where the continual transportation and aggradation of sediment material^3^ maintain a high level of geomorphic diversity. Temporal variability in transport rates and channel morphology is a fundamental element of a braided river mechanism. Multi-thread river systems with an intensive flow regime are prone to reoccupation of abandoned channels and relocation of bars in the river’s active zone^4^. Avulsion due to channel shift to the lower part of the floodplain is considered a sign of lateral channel instability^5^ exceeding the equilibrium thresholds. Non-cohesive gravel deposits in wide braidplains with frequent and extreme variations in channel depth create during flood events the proper conditions for sediment wave formation^6^. This typical longitudinal sediment connectivity considered as a sediment pulse (e.g.^7,8^) provides natural settings in river system behavior and adjustment. Due to anthropogenically influenced disruption of the natural development of river channels, there is a certain characteristic sequence of morphological changes of the riverbed in the process of adaptation to changed conditions as a critical barriers for landform modification^9^. The history of disturbance events determines the length of the evolutionary record that must be evaluated to assess the timing, rates and extent of geomorphic adjustments to disturbances, and whether these are expected, accelerated, suppressed or anomalous^10^. The state of former river systems in the European region over the past century reflects mainly progressive human impact (gravel mining, river training, small hydropower plant construction, reforestation, etc.) contributing to significant morphological changes mostly represented by channel incision and narrowing (e.g.^11–16^). In terms of hydromorphological assessment, the Water Framework Directive (WFD) proclaims indicators and criteria for the significance of their impact^17^, which interpret the disruption of a river system’s lateral and longitudinal continuity. Additionally, according to the WFD, river management requires maintenance or improvement of the ecological state of rivers. It is worth emphasising, though, that an improvement of the available EU guidances should be more focused on the causal interrelationships between hydromorphological alterations, sediment transport and the biological (ecological) status of freshwater ecosystems^18^. Moreover, Directive 2007/60/EC on the assessment and management of flood risks (entered into force on 26 November 2007) shall be carried out in coordination with the WFD. The fact that using the high-resolution multispectral images and topographic data at the national scale across Europe already reached a good level of detail, sufficient to support hydromorphological assessment for WFD^19^ corresponds to the methodological basis of our investigation on the Belá River. With a view to giving rivers more space in sense of the nature-based solution concept^20^, municipalities involved as decision makers should consider, where possible, the maintenance and/or restoration of floodplains recommended by Solín^21^ within decentralization and diversification of flood risk governance in Slovakia. Furthermore, in respect of adopting a holistic approach to promoting the sustainable development of aquatic ecosystem management^22^, a new assessment framework in a river basin context (RBWSI—River Basin Water Sustainability Index) has been developed^23^. Consequently, equivalent process‐based approaches to river management have been applied elsewhere in the world (e.g. ^24,25^) which gives us at least a high presumption of success in implementing similar approaches in our country as well.

The work presented here is analyzing the example of environmental change of multi-thread river channel with external disturbances represent by river training as a main factor influenced its morphological response. The present study was conducted in the Liptov Basin, located in the north of Slovakia, which contains the longest multi-thread river in Slovakia. This paper evaluates how the engineering approach to river training has influenced development of the hydromorphological complexity of the Belá River after the flood event in July 2018. On 11 river reaches (RR) where the management intervention was applied, we focused on the following quantitative indicators: (1) changes in the planned and realized length of intervention; (2) spatial and temporal variation in channel morphology; (3) changes in cross-section measurements; (4) changes in hydraulic parameters. The research hypotheses are related to decreasing geodiversity in managed river reaches, a rapid increase in flow velocity during an extreme flood, and increasing erosive force in the channel zone. Identification of main conflicts of the Belá River management is important for clarifying the different approaches of stakeholders in the study area and aims to provide an objective illustration of their consequences.

## Study area

**Figure 1:**
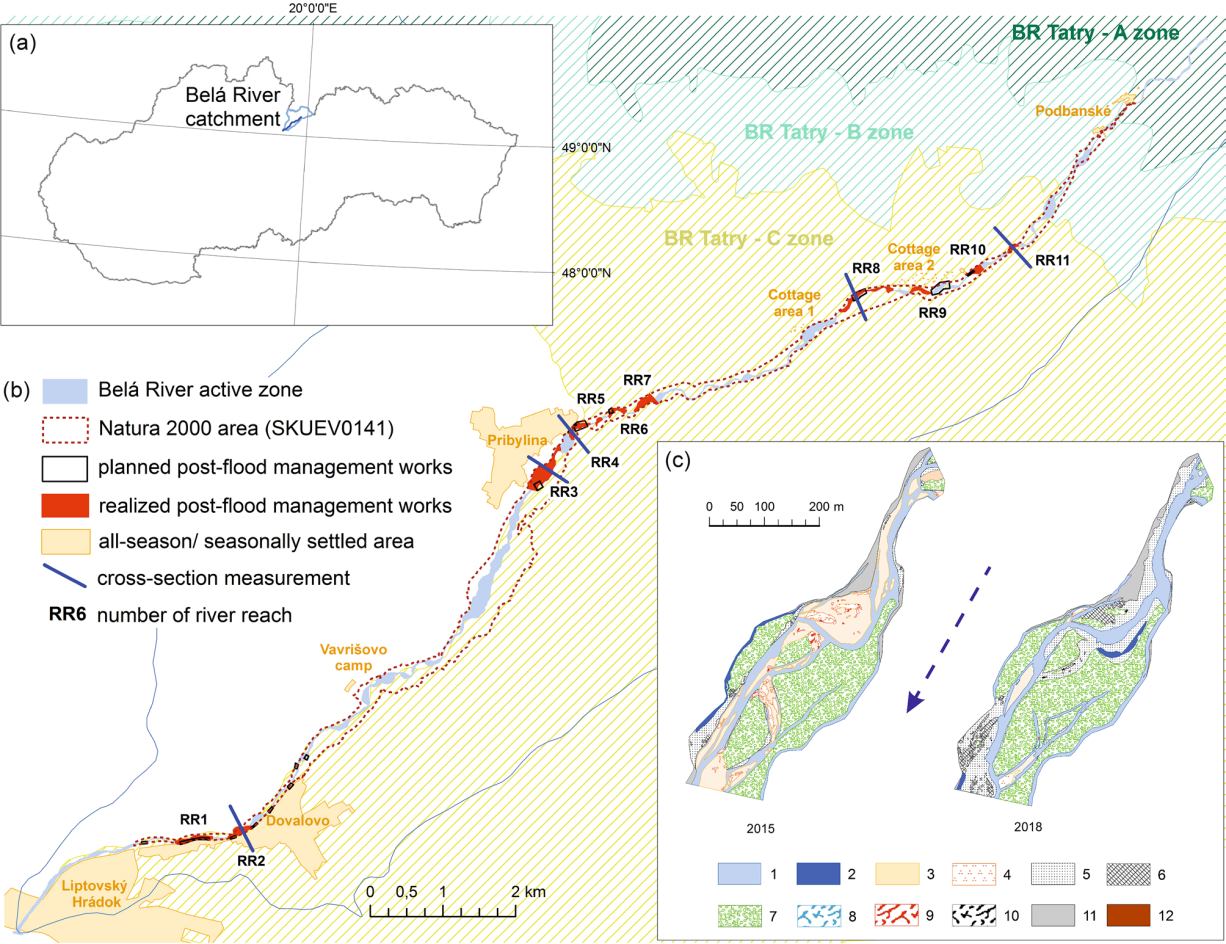
(**a**) Study area location in the north of Slovakia. (**b**) Spatial distribution of modified river reaches (sediment material transfer and extraction, transfer of heavy equipment) and repeated cross-section measurements in the Natura 2000 area of the Belá River (SKUEV0141). (**c**) Vectorized morphological channel parameters for the example of RR3 pre- (2015) and post-flood management intervention (2018), where 1—low flow channel area; 2—backwater; 3—bare mid-channel bar area (without vegetation); 4—mid-channel bar area with herb and shrub vegetation cover; 5—bare lateral bar area (without vegetation); 6—lateral bar area with herb vegetation cover; 7—island area with tree vegetation cover; 8—large woody debris (LWD) area in low flow channel; 9—LWD area on the mid-channel bar; 10—LWD area on the lateral bar; 11—floodplain area; 12—artificial structure area. Dashed arrow represents flow direction. This figure was generated in ArcMap 10.3 software.

The Belá River represents a unique morphological and ecological river body located in the foreground of a glacially modelled river basin in central European conditions in Slovakia (Fig. 1A). Thanks to its multi-thread planform, it represents a typical gravel-bed braided and wandering river^26^ with a length of 14.92 km^27^. A natural process by the rapidly changing flow regime increases the transport of large amounts of sediment and frequent relocation or creation of new gravel bars and arms (avulsions) on the adjacent floodplain^14,15,27,28^. During high flood discharges, there is an important geomorphological effect of the connectivity of the Belá River braidplain with the floodplain and the riparian zone. This type of hydrological connectivity of the entire river system is particularly important from a geomorphological point of view for maintaining the natural erosion–accumulation processes typical for high-energy rivers, to which the Belá clearly belongs^29^. In addition, a laterally connected braidplain with riparian forest on the floodplain can fulfil a natural flood protection function during floods (i.e. flood wave mitigation). Of course, the supply of sediments, nutrients and organisms also plays a major role. From an ecological point of view, the Belá River creates specific habitats bound to the often-disturbed habitats of gravel bars in the river’s active zone (e.g. habitat of *Myricaria germanica*), riparian flora or aquatic fauna (e.g. Natura 2000 species). The Belá River together with its riparian zone creates a biocorridor (§2, letter e), Act no. 543/2002 Coll. about nature and landscape protection, National council of the Slovak Republic) of supra-regional importance and, thanks to habitats of European importance (SKUEV0141), an area of 315.655 ha is included in the Natura 2000 Network^30^ (Fig. 1B). Despite some human activity affecting, the Natura 2000 protected area (flood protection dikes, bank stabilization near road, small hydropower plant operation), the Belá has retained a near-natural multi-thread character along with some river segments^15^. The Belá River basin has been included by the Slovak Hydrometeorological Institute (SHMI) in the international category as a representative river basin and is registered within the UNESCO International Hydrological Program^31,32^. The 16 km of the Belá WILDRiver was the subject of a European Wilderness Society Quick-Audit in summer 2017 and met the Bronze Wilderness Quality Standard (https://wilderness-society.org, European Wilderness Network). Additionally, the Belá River from the confluence with the Váh River to the confluence of Kôprovský and Tichý creeks is part of all three zones (A, B, C) of the Tatry biospheric reservation (BR). Most river management activities are in line with the official Slovak Water Management Enterprise (SWME) policy focus mainly on flood protection issues.

The cool and humid air mass flowing into the Carpathian region from north to northeast caused intensive atmospheric precipitation during 17–22 July 2018. The maximum daily precipitation and maximum discharge at Podbanské gauging station were 35.8 mm and 85.9 m^3^ s^−1^ respectively, which caused a flood situation with a 5-year recurrence interval (RI). At Liptovský Hrádok gauging station, the maximal discharge of 124.5 m^3^ s^−1^ reflected a 5–10-year flood. This flood event reached the third degree of flood activity at both gauging stations^33^ and was announced as a flood risk by water authorities. The flood event on the Belá River resulted in bank overflow at several sites, crossing the floodplain and road in one case, and intensive lateral bank erosion near the holiday cottage area with one cottage at real risk (Fig. 3E). Technical realization of the river training performed by the SWME within the braidplain of the Belá River according to the official technical documentation consisted of the following steps: (1) release of obstructed cross-sections; (2) relocation of sediment material (alluvial gravel bars) with a total volume of 46,600 m^3^; (3) removal of large woody debris (LWD) including its root system; (4) repair of damaged flood protection dikes and stony grade-control structures by refilling a total volume of 2050 m^3^ with quarry stones over 500 kg; (5) relocation of a total volume of 24,000 m^3^ of sediment material within the riverbed; (6) changing the streamline of the main channel. All of these activities, specified as necessary interventions to ensure the functioning of the riverbed, aimed to implement legislative measures to prevent further post-flood loss.

## Methods

### Analysis of the impact of river training on river morphology

Based on very accurate remote sensing data (provided by Eurosense, s r.o.) with a resolution of 20 cm pixel size, from reference conditions in 2015 in the pre-flood management period (imagery date: July 7, 2015) and conditions in 2018 in the post-flood management period (imagery date: August 12, 2018) and field reconnaissance, the planned and realized river training were quantified. Morphological changes to the river’s active zone were quantified and evaluated. Nine categories of morphological parameters present in-channel landforms with different types of vegetation cover were studied: (1) water area, length of low flow channels; (2) isolated water area (backwater); (3) lateral gravel bar area without vegetation; (4) mid-channel gravel bar area without vegetation; (5) bar area with vegetation cover (herbs and shrubs); (6) island area (tree vegetation); (7) large woody debris area in low flow channels; (8) LWD area in the mid-channel bar; (9) LWD area in the lateral bar (Fig. 1C). The nine morphological parameters were chosen according to the possibility of their detection on the ortophotos and according to their relevance for the morphological in-channel landforms changes comparison as well as identification of the river pattern changes. Artificial channels formed in the river’s active zone by the accumulation or relocation of gravel in the form of broad and elevated lateral bars at least to the level of the floodplain or even above it were designated as artificial bar surfaces. In the GIS environment (ArcMap 10.3), based on visible artificial change in braidplain area (i.e. visible traces of heavy equipment interventions) 11 river reaches belonging to the Natura 2000 area were indentified and vectorized on the Belá River.

The shapefile formats of all monitored river reaches, classified into the nine channel landform categories (i-ix), served as input data for the calculation of Shannon diversity index (aiming to prove changes in geomorphic diversity) performed with R software^34^ and the “vegan” ecology package version 2.5-6^35^.

### Cross-section measurement and hydraulic parameter modelling between pre- and post-flood management periods

The fieldwork campaigns focused on capturing changes in elevation at five cross-sections (RR2, RR3, RR4, RR8, RR11) in artificially modified river reaches (RR). The changes in cross-section measurements were determined with a GPS Leica Zeno5 with RTK corrections and vertical and horizontal accuracy of 30–100 mm for all consecutive cross-section measurements assigned to the period 2017–2019. The pre-flood management period (2017–2018) before the flood in July 2018 was utilized to capture naturally occurring erosion–accretion processes on expertly selected cross-sections on the studied river reaches. The post-flood management period (2018–2019) after the 2018 flood and after heavy equipment interventions in the river’s active zone was used for further cross-section measurements in August 2018 and July 2019.

To calculate the width/depth (*W*/*D*) ratio, the bankfull channel width was acquired directly from the cross-section data collected in the field. Thus in each cross-section, bank edges were indicated at the place of a first sudden change in the cross-section profile, above which the surface was covered by permanent vegetation^36^. It allowed the model to partition a total flood flow into the flows conveyed in the channel and floodplain zones of the cross-section and to compute given hydraulic parameters not only for the total cross-section but also for its channel and floodplain parts. Bankfull channel depth was calculated as the average of all measurements taken at the cross-section in each sampling site.

T-years flood discharges within the study cross-sections (RR2, RR3, RR4, RR8, RR11) were calculated using the information obtained from Liptovský Hrádok gauging station about values of t-years floods there and additionally calculations of the catchment area in the vicinity of study cross sections. For discharge value extrapolation the formula giver by Ozga-Zielińska and Brzeziński^37^ was applied:

1 $$Q_{X\max } = Q_{W\max } \left( {\frac{{A_{X} }}{{A_{W} }}} \right)^{n}$$

where *Q*_*X max*_—discharge at the measured cross-section (m^3^ s^−1^); *Q*_*W max*_—discharge at the gauging station (m^3^ s^−1^); *A*_*X*_—catchment area for the measured cross-section (km^2^); *A*_*W*_—catchment area for the gauged cross-section (km^2^); *n*—parameter of the extrapolation equation. Manning’s roughness coefficients for the channel bank and floodplain parts of the stream cross-sections were chosen in line with the criteria of Chow^38^ and are changing along cross section. Also Manning’s roughness coefficients they are different for pre- and post-management period analyzed in the paper.

The following hydraulic parameters for the measured cross-sections with potential RI (from bankfull to Q100) were analyzed in the channel zone: flow velocity (m s^−1^), shear stress (N m^−2^) and stream power (W m^−2^). The constructed hypotheses relate to the impact of river training on the flow velocity for the new artificial geometry of the main channel. We assumed that a new single-thread channel configuration with artificially high banks increases flow velocity significantly. Shear stress is a measure of the erosive force of running water in a riverbed. In comparison with the original morphological configuration of the main channel, higher values were anticipated as a negative influence of management intervention on shear stress values. For stream power, an increasing trend of erosive capacity concurrent with increased flow velocity and erosive force is presumed. Measurement of the *W*/*D* ratio^39^ is valuable for describing channel cross-section shape, and comparison of ratio values can be used to interpret shifts in channel stability following disturbances to channels. These analyses highlight the role of the *W*/*D* ratio on the degree of asymmetry of the discharge distribution in the channel. A one-dimensional, steady flow HEC-RAS model in line with Chow^38^ and Chang^40^ postulates (where the flow velocity, energy as well as other hydraulic equations are presented in details) was used^41^ to determine all analyzed hydraulic parameters as well as water stages for given flood discharges in the research cross-sections of the Belá River. String models were created for cross-sections, where the hydraulic gradient was calculated based on cross-sectional geometry, elevation of successive cross-sections and the distance between them. A mixed-flow regime mode was used for the modeling. The discharge data were taken from at two water gauging stations on the Belá: the upstream Podbanské gauging station and the downstream Liptovský Hrádok gauging station where the stage–discharge curves were available. Model calibration was done using the findings of Brandimarte and Baldassarre^42^ and USACE^41^.

## Results

### Geomorphological impact of the management interventions

Based on the official documentation of the SWME, the total length of the planned river training on the Belá River in the Natura 2000 area was 1340 m. GIS analysis proved, that the total length of the realized river training carried out in the form of relocation and extraction of sediment material was 4823 m. Due to individual assessment of the channel condition during the constructive works in the field, the official plan was carried out beyond its scope. These facts confirm that the total length of realized works exceeded the planned works by 360%. However, the appropriateness of these excessive interventions in realized form remains disputable.

**Figure 2:**
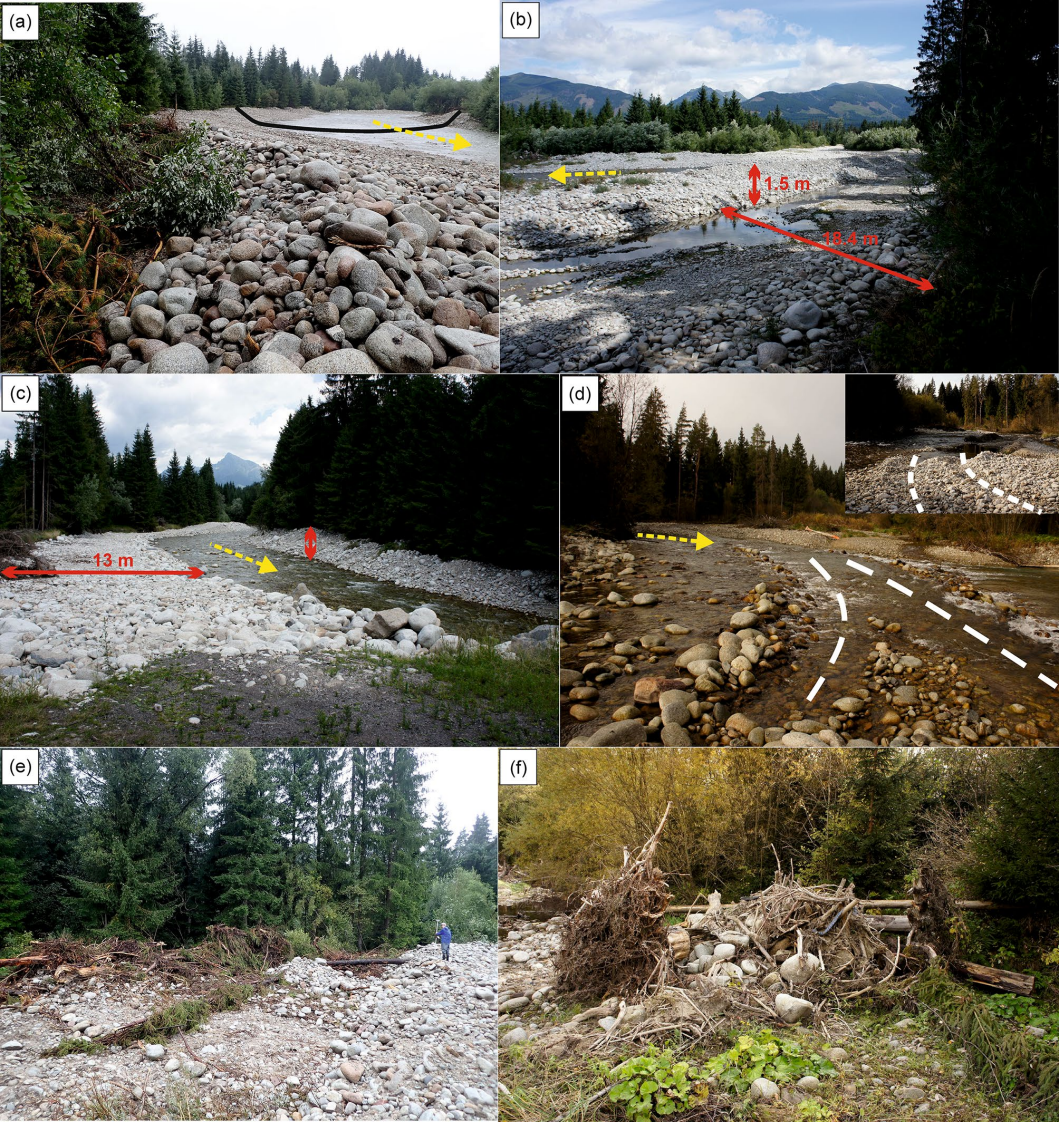
(**a**) Photographic documentation of how gravel has formed into a wide “U”-shaped channel and (**b**) of the artificial formation of backwater surfaces and elevated gravel barriers in the middle of the braidplain area near Pribylina settlement during field reconnaissance in 2018 and 2019. (**c**) We recognized the formation of artificial lateral bar surfaces and elevated gravel banks (marked with red arrows) further upstream at Kokavský Bridge in RR10. (**d**) Visible traces of heavy equipment transfer (white dashed lines) at two sites between the individual treated river reaches between Vavrišovo and Pribylina settlements. (**e**) On the edges of riverbanks, we documented the rolling up of large woody debris mass (**f**) mixed with granite boulders. Yellow arrow represents the flow direction (photo: Anna Kidová; A, E, F: 13.8.2018; B, C: 17.7.2019; D: 19.10.2018).

During field reconnaissance, several characteristic examples of the management interventions were found in the Belá braidplain area (Fig. 2). The river training in RR7 consisted of extraction or shifting of gravel to form a wide “U”-shaped channel with high banks, i.e. forming a shallow, quasi-artificial channel (Fig. 2A). By carrying out these management interventions, the original multi-thread river system was seriously or completely destroyed and the water flow was concentrated in a single shallow main channel. Upon the intervention of heavy equipment, unnatural backwater surfaces with stagnant water arose on the artificial bar surfaces in the river’s active zone (e.g. RR3, Fig. 2B). The next management intervention example is represented by shifting gravel into the high banks (Fig. 2C). Uplift of the original left bank line isolated it from the other sidearms on the floodplain. At the same time, a 13 m-wide artificial bar surface was created where the original 5.2 m-wide lateral bar occurred in the pre-flood management period in RR10. Orthophotos from 2018 as well as field reconnaissance also revealed well-marked traces of heavy equipment transfer between the individual river reaches in the Belá braidplain (Fig. 2D). The extent of bottom damage in overall 12 recorded river sites is only indicative, but certainly not negligible. The river training in the Belá River also included the removal of LWD or rolling of LWD along the banks or on bars (Fig. 2E,F). LWD residues in the braidplain created wood accumulations (clumps and jams) or were dispersed at irregular intervals as individual pieces.

**Figure 3:**
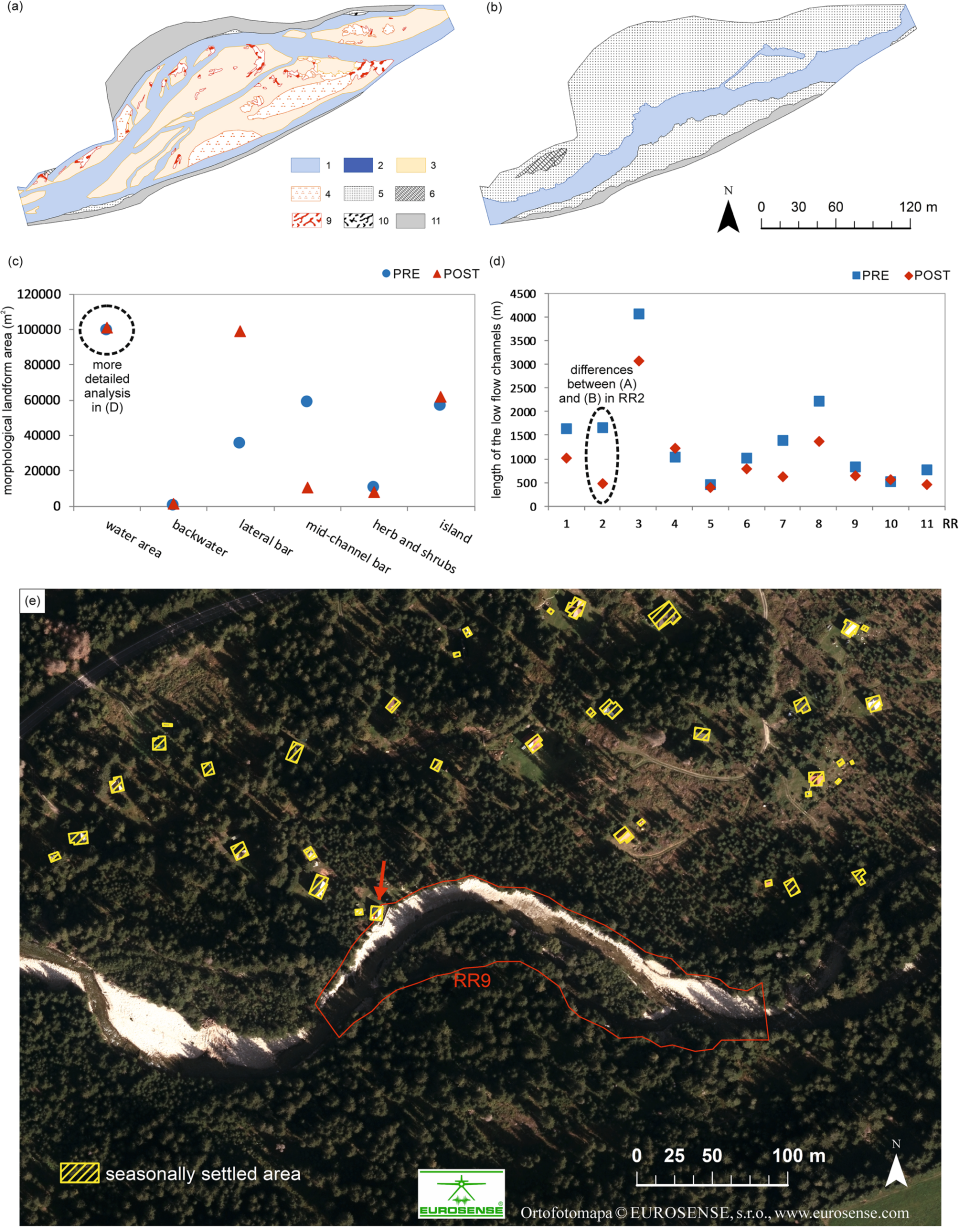
(**a**) Example of reduced low flow channels on RR2 near Dovalovo settlement in 2015 with natural braidplain before management intervention and (**b**) in 2018 after intervention with an artificially created lateral bar on both banks of the Belá River generated in ArcMap 10.3 software. (**c**) Comparison of area of studied morphological parameters in total for all studied 11 river reaches in the natural (2015) and artificial river active zones after river training (2018). (**d**) Differences in the length of low flow channels between 2015 and 2018 in all studied river reaches (RR1–11). For legend of studied channel landforms for (**a**), see Fig. 1C. (**e**) Holiday cottage at real flood risk (marked by a red arrow) in the seasonally settled “Cottage area 2” (Fig. 1) on the Belá River floodplain. This risky position of the cottage probably requires management intervention in RR9. Background ortophoto image is from 2018 and was generated in ArcMap 10.3 software.

Based on a comparison of the area and length of selected morphological parameters (Fig. 3C,D), several changes in the morphology of the Belá River’s active zone were evident. Although the results indicate that there has been no significant decrease in the water surface area (low flow channels) after the river training, the data on the length of the low flow channels, which refine these results, showed the opposite. After adjustments, the length of the flow channels decreased overall by almost 5 km. The decline was due to the shifting of gravel to the sides, destroying the original multi-thread pattern and creating a wide, single-thread one (Fig. 3A,B). The area of artificially created isolated water surfaces (backwater areas) on lateral bars was increased 30 times. There was a noticeable increase in lateral bar area overall due to the accumulation of gravel from the center of the main channel to its edges and to the banks, respectively. The area of the mid-channel bar, the most important and dynamic in-channel landform of a braided-wandering river system, decreased significantly after management intervention, from 59,015 to 10,313 m^2^. This intervention in the braidplain area resulted in a decrease in the bar area with herb and shrub vegetation cover as well, from 10,435 to 7881 m^2^. A slight increase in island area indicates the maintained continuity of vegetation succession.

**Figure 4:**
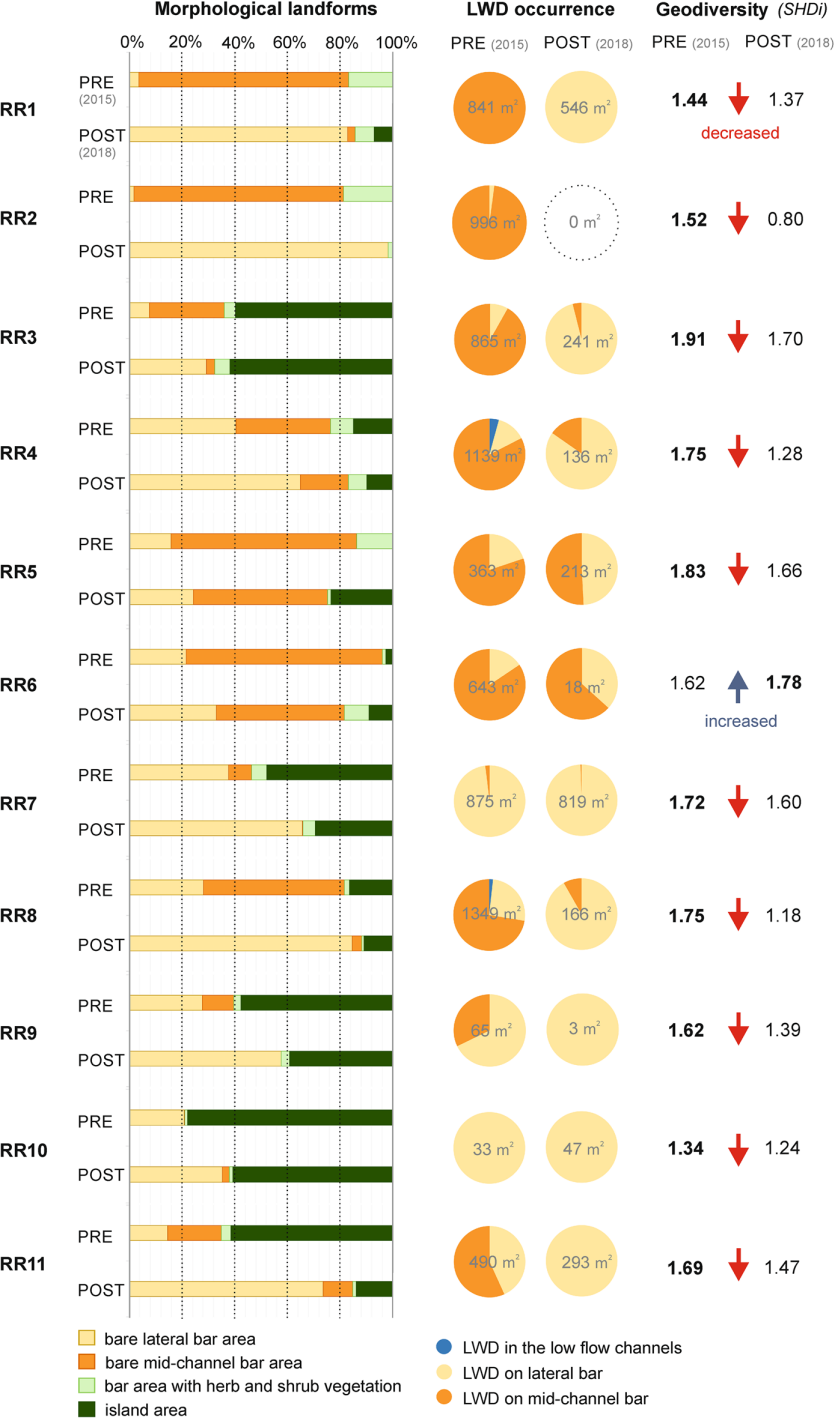
Differences in the morphological landform area of the braidplain, geodiversity status (increased/ decreased SHDI) and comparison of LWD accumulation area in PRE-flood (2015) and POST-flood management periods (2018) in all studied river reaches (RR1–11). Numbers inside a pie chart represent the total area of LWD accumulation identified in monitored river reaches.

The four main morphological landforms of the braidplain, bare lateral/mid-channel bars, bars with herb and shrub vegetation cover, and islands, were evaluated for all studied river reaches (Fig. 4). A specific quantifiable parameter, the presence of LWD in the braidplain area, was monitored especially in the low flow channels and on lateral and mid-channel bar landforms (Fig. 4). The equal scenario, with a significant decrease of the mid-channel bar area as well as a significant increase of the lateral bar area on all river reaches (RR1-9, RR11), prevailed. On a prevailing number of river reaches (RR1, RR2, RR4, RR5, RR11), vegetated bars with herbs and shrubs decreased in area after river training. The original island area occurred on seven river reaches in 2015. On six river reaches (RR4, RR7, RR8, RR9, RR10, RR11), island area decreased and only on two did it increase in 2018 (RR3, RR6). On two river reaches (RR1, RR5), a brand-new island area arose after management interventions.

The presence of LWD accumulations reduced significantly after river training in the Belá braidplain, from 7666 to 2482 m^2^ in total. We noticed a very significant decrease in LWD especially in mid-channel bars (from 5676 to 168 m^2^ in total), of which the lateral ones were artificially created. However, we registered only a slight increase (about 395 m^2^ in total) in LWD between 2015 and 2018 on lateral bars as well. LWD occurrence in 2018 prevailed in lateral bar areas (RR1, RR3, RR4, RR7-11) in comparison with the prevailing occurrence in 2015 on mid-channel bars. On two river reaches (RR4, RR8), we registered natural occurrence of LWD also in low flow channels. Calculation of geodiversity status (Shannon diversity index) proved a decreasing trend in geomorphic diversity for 10 river reaches after river training. We registered the largest difference (decrease) in geodiversity status between pre- and post-flood management periods mainly for RR2 (− 0.72) and RR8 (− 0.57).

### Morpho-hydraulic dynamics in cross-sections

**Figure 5:**
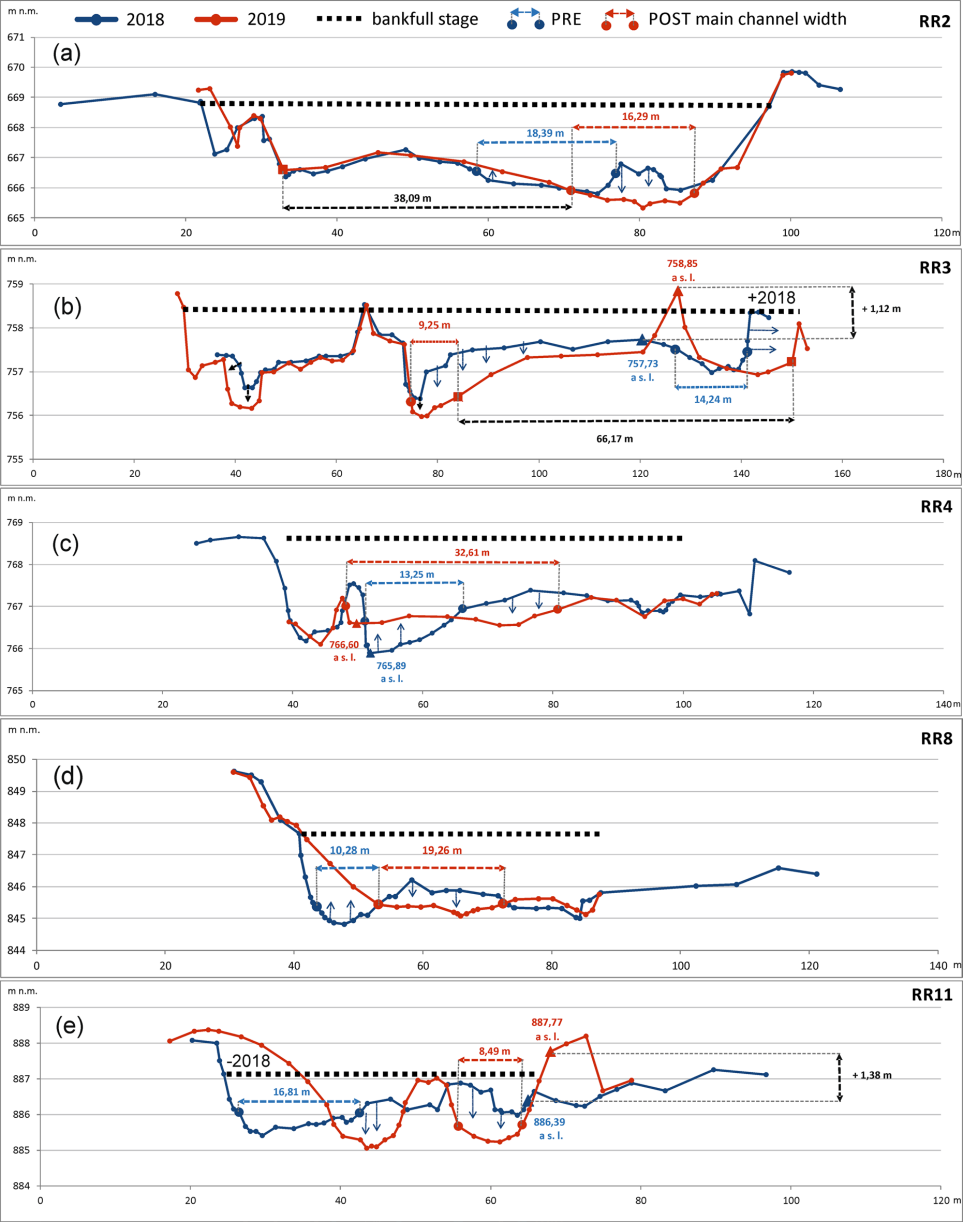
Geometry of cross-sections on the selected river reaches (**a**) RR2, (**b**) RR3, (**c**) RR4, (**d**) RR8, (**e**) RR11 on the Belá River where river training were carried out. The blue cross-section represents the reference state (PRE) of the river before the flood in July 2018; the red cross-section represents the state after this flood event and river training during August 2018 (POST). At each cross-section, the width of the main channel (m) is marked (red and blue numbers). Bankfull stage at each cross-section reflects measured bankfull width where value ± 2018 points out differences in width between pre- and post-flood management periods. The direction of the arrows marked in cross-sections represents surface modification of the river’s active zone during river training.

Repeated individual cross-section measurements after river training and modification of the Belá River braidplain revealed significant reductions in the flow depth in all cross-sections (Table 1, Fig. 5). The morphological structure (bar, island, low flow channels, artificial bar surface) of these cross-sections are summarized in Table 1 for the pre- and post-flood management periods.

**Table 1:**
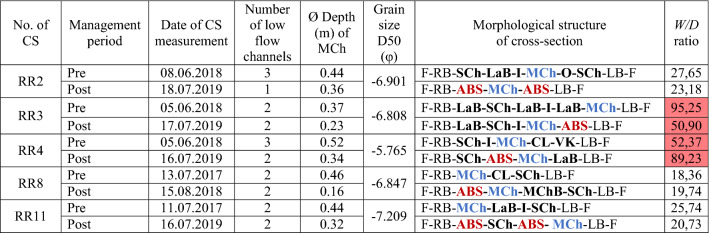
Selected morphological parameters (number of low flow channels, depth of main channel), morphologic structure (in the right to left order) and *W/D* ratio of monitored cross-sections on river reaches where the river training was realized (Pre: before the flood event in July 2018; Post: after the flood event in July 2018 and management intervention).

Median grain size (D50) of the sediment material was measured on gravel bars as well as under the water on the main and secondary channels based on Wolman^43^ approach.

*CS* cross-section, *RR* river reach, *F* floodplain, *RB* right bank, *LB* left bank, *MCh* main channel, *SCh* secondary channel, *LaB* lateral bar, *MChB* mid-channel bar, *I* island, *ABS* artificial bar surface).

The first cross-section RR2 (Fig. 5A) is situated in the village of Dovalovo (urban part of the town of Liptovský Hrádok). The original multi-thread pattern was destroyed across its entire width. The original main channel was transferred from the central part to the left side of the braidplain, while two original island landforms were removed. The width of the artificial bar surface was 38.09 m, representing two-thirds of the total width of the river’s active zone at this cross-section.

Cross-section RR3 (Fig. 5B) is located in the settlement of Pribylina. On the left side of the river’s active zone, a 66.17 m-wide artificial bar surface was formed. The top of this artificial bar surface (in the form of a dike) exceeds the original top of the mid-channel bar by 1.12 m (Figs. 2B and 5B). This cross-section is further characterized by a reduction of the erosion base of both the main and side channels by about − 0.5 m. The position of the main channel was artificially shifted to the center of the braidplain, reducing its width and depth.

The third cross-section RR4 (Fig. 5C) is situated under the road bridge between the settlements of Pribylina and Liptovská Kokava. At the cross-section, the bottom of the main channel was levelled and raised by 0.71 m. The main channel was artificially extended from the original 13.25–32.61 m, while the original mid-channel bar was removed.

Cross-section RR8 (Fig. 5D) is located near the right-side tributary of the Bystrá creek to the Belá River. At this cross-section, on the right side of the river’s active zone, there was massive accumulation of gravel material and filling of the original 10.28 m-wide main channel. New artificial 19.26 m-wide and shallow (0.16 m average depth) main channel was created (Table 1).

Cross-section RR11 (Fig. 5E) is located upstream on the Belá River, above the Kokavský Bridge site. Piled-up gravel on the right bank caused destruction of the original 16.81 m-wide main channel. By adding gravel on the left bank, the original bank height increased by 1.38 m. A new 8.49 m-wide main channel was artificially created on the right side of the river’s active zone. The management intervention raised the riverbanks, notably increasing the isolation of the main channel from the surrounding floodplain.

The cross-section measurements of the modified river active zone on selected river reaches (RR2, RR3, RR4, RR8, RR11) clearly demonstrate several negative effects on the original morphology of the Belá River. We registered simplification of the multi-thread river pattern, artificial shifting of the main channel’s position and the formation of uniform morphologically undifferentiated anthropogenic forms (artificial bar surfaces) in the river’s active zone, as well as isolation of the main channel from the surrounding floodplain by artificially raised banks. The changes in cross-section geometry caused by the management disturbance were also naturally reflected in the values of the *W*/*D* ratio. We registered a different *W/D* ratio for pre- and post-flood management periods for all measured cross-sections. These values proved the most significant asymmetry of the river’s active zone in the discharge distribution in cross-sections RR3 and RR4 (Table 1).

**Figure 6:**
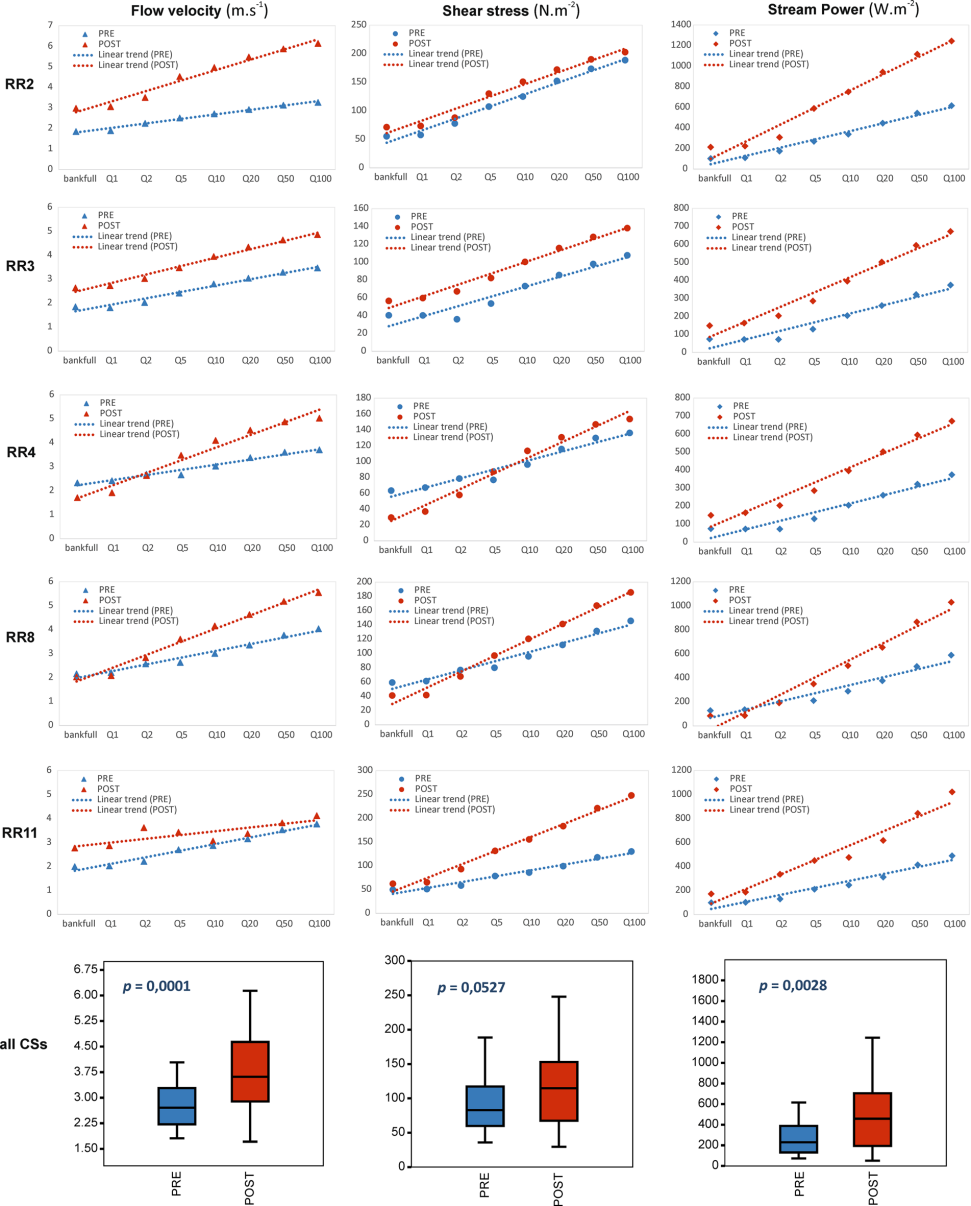
Hydraulic parameters calculated in measured cross-sections (CSs).

Average values of hydraulic parameters for the five studied river cross-sections at eight flood discharges of given RI (bankfull, 1-, 2-, 5-, 10-, 20-, 50- and 100-year) were compared and the statistical significance of differences between two types of cross-section (pre- and post-flood management period) was determined (Fig. 6). For the first hydraulic parameter monitored, an increase in flow velocity in the modified main channel was documented for all cross-sections. Overall differences in flow velocity in all cross-sections were significant according to the Mann–Whitney test (*p* = 0.0001). For the shear stress parameter, we documented a clear increase in erosive force from 5- to 100-year RI in all cross-sections in the post-flood management period. Overall differences in shear stress in all cross-sections were on the borderline of significance according to the Mann–Whitney test (*p* = 0.0527). Analysis of the stream power parameter demonstrated its higher values of the erosive capacity of sediment transport during the post-flood management period in all cross-sections. The much intensive erosive capacity shows in higher RI values mainly, hence the much more eroded material during high flood events is expected. This trend reflects the significance value of the Mann–Whitney test as well (*p* = 0.0028). The higher amounts of transported sediments support the accumulation zones increasing as well.

## Discussion

### Morphological and hydraulic impact of the management intervention

The management intervention in the Belá River’s active zone was demonstrated by the creation of wide “U”-shaped shallow channels, artificial bar surfaces (lateral bar areas) and high banks. New (artificial) morphological landforms of accumulated LWD mixed with gravel originated as well. The passage of heavy equipment through the riverbed used to move from one planned treatment river reach to another caused disturbance of the river bottom and deformation of gravel bars. Its impact on the bottom cohesion of the Belá River increased the water energy, i.e. its drifting ability, thus increasing the risk of channel incision^12^ into the easily erodible flysch bedrock (sandstone and claystone). Reducing the erosion base (by incision) can disrupt or even completely disrupt the hydromorphological connectivity of the main channel with the secondary channels in the transverse direction. We ascribed a similar effect to the formation of high banks. The functional, structural and hydrologic connectivity^44,45^ between the channels in such a floodplain is important mainly to preserve the typical multi-thread pattern of the Belá River. Vehicles crossing the channel resulting in the destruction of bed armoring has also been pointed out on the Raba River in the Polish Carpathians^46^.

The presence of LWD in the riverbed has a significant geomorphological effect^47–49^, especially in reshaping in-channel morphology (e.g. sediment transport) as well as lateral migration of the riverbed (e.g. bank erosion, avulsion). By its size or position in the channel, LWD creates a barrier to flow and/or initiates trapping of finer sediments, accelerating vegetation cover formation processes^50^. It is highly probable that the unsystematic relocation of gravel and LWD on the edge of river banks and on gravel bars has led to disturbance and destruction of the original fauna and flora biotopes that are included in Natura 2000 (e.g. *Myricaria germanica*) due to eradication of the geomorphic features, such as pools and riffles^51^ and reduction of their bio-geomorphological function^52–54^. The riparian zone of the Belá River represents the best-preserved riparian forest stands with German tamarisk *Myricaria germanica* in Slovakia. The last biomonitoring carried out by The Slovak Nature Conservancy in 2019 revealed that this biotope is currently in 25% of the inconvenient state, in 50% of bad conditions, and only in 25% of the favorable state due to management intervention in 2018. The irregular and unsystematic relocation of gravel with LWD outside of the river’s active zone or its removal from the mid-channel part to the nearest bank destroyed the original biotopes of fauna (macro- and microfauna). The results of the inventory survey on the Belá River carried out by the Slovak Fishing Association, after river training in October 2018, confirmed damaged fish biotopes with affected species composition, abundance, and the age structure of the ichthyofauna. According to the recorded results, the estimate of fish mortality due to the implemented flood control measures reached 15–20%. Besides that, the new position of LWD near bank lines could lead to greater bank stabilization due to the bank armoring effect.

The actual global problems of climatic change^55,56^ related to water deficiency (drought) or flash floods, as well as decreasing bio- and geodiversity^57,58^, are alarming for society and have an impact on many decisions and attitudes of policymakers. For river systems in the European region, the WFD (2000/60/EC) represents the basis for river management optimization and the focus of its ecological quality improvement activity until 2027. The WFD in Annex II requires the identification of significant morphological changes in water bodies. Elements defining morphology include variation in the depth, width, structure and substrate of the riverbed and structure of the riparian zone. Disturbed natural river morphology affects the habitats of aquatic plants and animals and can therefore have an impact on aquatic ecology. The ability for adaptation and flexibility in river management is necessary for the sustainable development^59^ and protection of unique river landscapes.

We demonstrated changes in channel morphology and flow hydraulics by hydraulic simulation for the studied river cross-sections. As far as uncertainty of numerical modeling is concerned, hydraulic conditions in rivers are usually complex flows but they can be analysed with use of 1D or 2D modelling^60–62^. Whilst 1D approach is often considered a gross simplification of the flow field^63^, one can justify the approach by assuming that the approximations involved in treating out-of-bank flow as one-dimensional are small compared to geometry in the incised and multi-thread reaches^64,65^. As demonstrated by Horritt and Bates^61^ for a 60 km reach of the River Severn, UK, one-dimensional models performed equally well. Importantly, the approach allowed us to obtain the detailed river geometry in the studied cross-sections and to extend the study to a completely considered river reach. The chosen flood magnitude discharges (from bankfull to 100-year RI) more precisely reflect the whole scale of the potential morphological effect after management intervention. The documented increases in flow velocity, shear stress and stream power for all studied cross-sections on the Belá River (Fig. 6) reflect the river system’s response to the new morphological settings within the modified main channel (post-flood management period), which might result in accelerated erosion of the riverbed^39^. The increased values from bankfull discharge to 100-year RI of shear stress from 1.93 to 33.37% and stream power from 36.26 to 83.57% in average of all measured cross-sections correspond to reduced multi-thread channel configuration with mid-channel bars occurrence. The resulted artificial created shallow main channel with lateral bars along river banks decrease the width–depth ratio due to decreased width of the initial channel. Our findings confirmed arguments that the channels with large width–depth ratios will braid through the development of multiple bar-unit structures across the width of the channel, whereas constraints on adjustment in width–depth ratio result in meandering or even straight channels^66^.

The synergistic effect of management interventions such as channel straightening, pattern simplification or lateral connectivity disruption has led to a significant decline of geodiversity on the Belá River but also to reduced ability of the river system to mitigate flood discharges. In the Carpathian region, increased values of shear stress, as well as stream power, have been documented on managed river reaches in comparison with unmanaged ones on the Raba River in the Polish Carpathians^67^. Analyses of the stream power in the multi-thread cross-sections of the Czarny Dunajec River also revealed lower values in comparison with the channelized ones as well (from 3.3 times at a 1.5-year flow to 4.5 times at a 50-year flood)^68^. A similar trend in the Czech Carpathians proves increasing stream power values for higher-magnitude flow events (5-, 20-, 50-, 100-year RI) on the gravel-bed Olše River after channel transformation due to channelization^69^. Another confrontation with modelled flow hydraulics on the re-naturalized multi-thread patterns of the Bečva River refers to the detection of lower unit stream power values^70^. These findings clearly correspond with our hypothesis linked to channel adjustment in the post-flood management period. All research hypotheses were confirmed: flood wave mitigation on the floodplain was minimized and erosive force in the main channel increased. On the other hand, these facts in the context of flood risk brought unsatisfactory results.

### Identification of the main conflicts of the Belá River management

This paper aims to draw attention to the disharmony between the content of strategic documents and the engineering approach to river management for the protected area (Natura 2000) through the objectiveness of geomorphological and hydrological assessment. Nowadays, several studies reflect prioritizing an integrated and holistic approach^71–73^ before an engineering one. Another effective approach is represented by adaptive management^74,75^ based on the systematic process of simulation models to test hypotheses that reflect different interpretations of how river processes work. The official document of Slovakia’s water plan from 2015 for river basin management, a comprehensive tool for setting objectives and measures for the Danube basin in which the Belá River is located, extended the identification and analysis of the significance of transverse disturbances to connections of wetlands and inundations (floodplains) on all water bodies for the second plan period (2016–2021). Equivalent priorities, linked to the prevention of further deterioration of water status when planning new projects that may cause new hydromorphological changes in the body of surface water in accordance with the provisions of Article 4 (Section 7) of the WFD, are mentioned in another strategic document, the “Proposal of orientation, principles and priorities of water management policy for the Slovak Republic until 2027”. The orientation of water management policy refers to the use of green infrastructure and the opening/restoration of natural floodplains, especially in the zone outside municipalities, as one of the preventive measures for flood protection. On the contrary, solutions for river channels in the urban area of municipalities are linked to technical measures using so-called “grey” infrastructure, in particular, due to the limited space between the stream and existing buildings, where using the elements of “green” infrastructure is not possible or insufficiently efficient. As Kidová et al.^15^ pointed out, the Belá River has a near-natural character. From a management point of view, the spatial distribution of settlements along the banks of the Belá River is generally sparse. In the first 5 rkm from the confluence of the Belá River with the Váh River, there are the settlements of Liptovský Hrádok and Dovalovo, protected by dike construction and several stony grade-control structures. The next settlement on the Belá River is Pribylina, protected by bank reinforcement. The Vavrišovo camp and cottage area situated near the Hrdovo and Kokavský Bridge spot upstream on the Belá River is seasonally occupied by people, mainly for recreation purposes. Cottage area 2 (Fig. 1) represents management intervention along RR9 due to location of the cottage very close to the right bank of the Belá River (Fig. 3E). Flood protection is necessary in areas inhabited all year round as well as seasonally, for logical reasons. Although the cottage area is situated on the river terrace of the Belá, the human impact should be better managed in this area, for instance by restricting permits for the construction of cottages near the riverbanks. In contrast, the opposite left floodplain area provides sufficient room for flood overflow discharges and represents a suitable river reach in terms of the “room for the river” concept^76^.

Evidently, the hydromorphology of the Belá River as part of the Natura 2000 protected area has changed as a result of post-flood management intervention in 2018 under the responsibility of SWME. In general, specific artificial bar surfaces were created and the braidplain was isolated from the floodplain. The proposed Natura 2000 management recommendations for the Belá River (SKUEV0141) relate to the revitalization of the braidplain by the restoration of abandoned channels and side arms for the purpose of wetland habitat (floodplain) inundation^30^. The principles of care for non-forest habitats of European importance such as fresh water habitats and their non-forest riparian vegetation are represented by preservation of the nature of the stream, including the amount and speed of water flowing in the riverbed, water quality (prevention of pollution and eutrophication) and preservation of unregulated river banks. In gravel-bed streams such as the Belá River, it is necessary to ensure the process of transformation or moving of gravel bars during floods and to create conditions for the preservation and restoration of the Br3 habitat (Mountain freshwaters and their woody vegetation with *Myricaria germanica*). Moreover, regulation of gravel extraction is necessary. The priority to maintain and improve the situation in the territory of the Belá River is low to medium. However, presented human interference on the Belá River is clearly in contradiction with the proposed management recommendations. Furthermore, the guidance document devoted to the identification and designation of heavily modified (HMWB) and artificial water bodies (AWB)^77^ interprets the management intervention performed on the Belá River as a significant anthropogenic pressure which disrupts the river continuum and causes alteration of the hydraulic regime due to straightening, as well as having an impact on its hydromorphology. According to all the above mentioned arguments, the post-flood management approach on the Belá River was chosen incorrectly. This evident non-compliance with the strategy documents has caused public nuisance for inhabitants in settlements linked to the Belá River and evoked discussion about the consequences and correctness of the chosen management procedure among experts as well as non-government organizations and ecological activists (Fishing Union, Association of Limnology, Association of Slovak Geomorphologists, World Wide Foundation), resulting in the preparation of a memorandum for their cooperation. After all, in summer 2018 after river training, the Belá WILDRiver was officially removed from the European Wilderness Network, interpreted as a practical consequence.

The lack of a linked decision process between stakeholders and research findings on the basis of geomorphological and sedimentological studies was evident in the Carpathian region two decades ago. The indication of weak points in the engineering approach to channelization pointed out by Wyżga^46^ refers to criticism of the general application of empirically derived formulas for bedload transport that may lead to significant errors in predicting the bedload transport rates in a channelized river and hence to its instability. Surian^11^, looking at examples of braided gravel-bed rivers in northern Italy, noticed that for river management it is necessary to recognize the effects of channel adjustments on structures and the environment as well as understand the causes of morphological changes.

**Figure 7:**
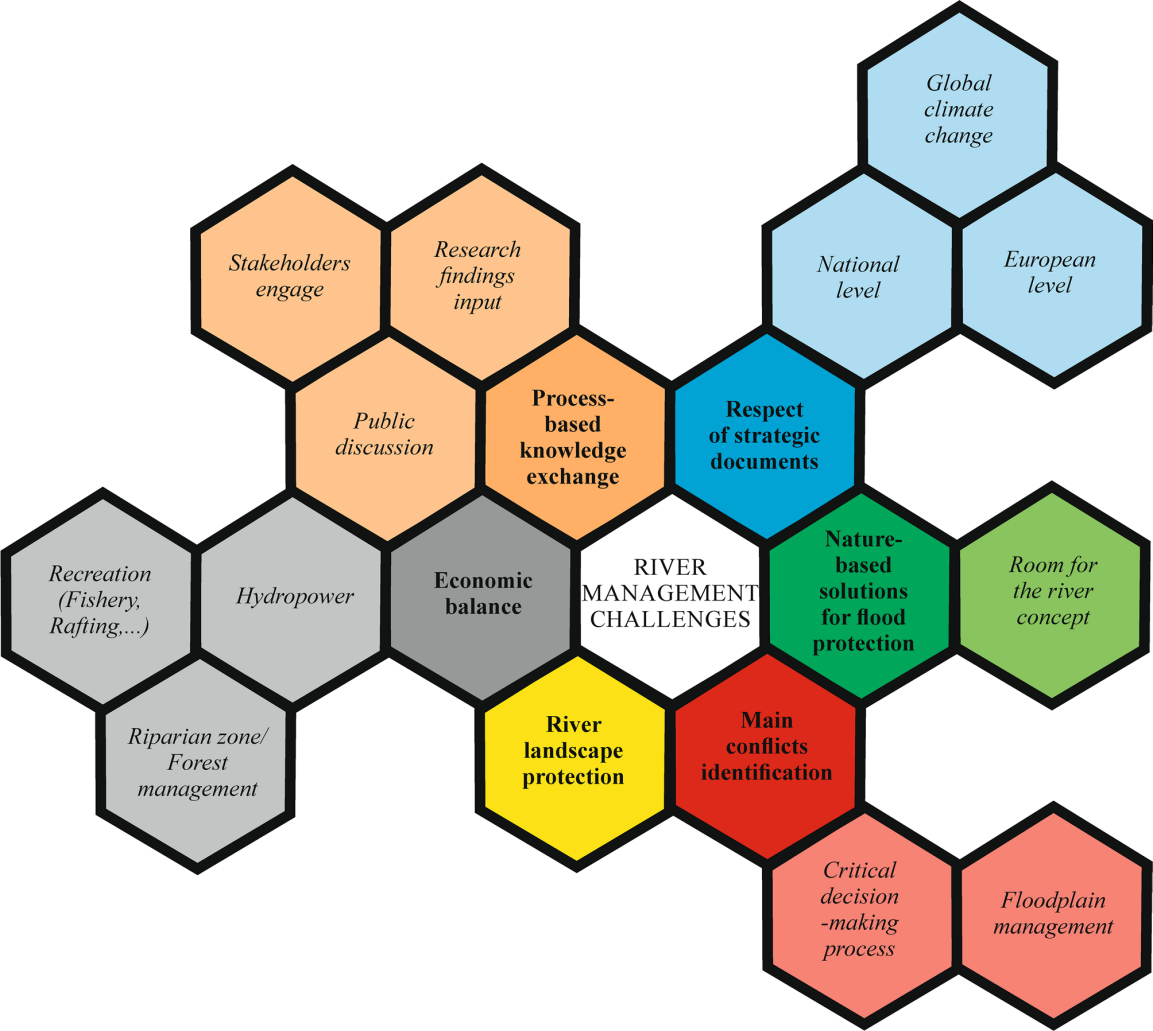
River management challenges scheme suggested by authors reveals the main areas for improvement the management strategy for the Belá River.

Existing documentation for management program of the Belá River for the period 2018–2047^78^ is focusing mainly on setting operational objectives in relation to ecologically functional zones represented by specific protected habitats of fauna and flora. The complex river management is missing at all. Hence, there is a strong need for integrated management and consensus between water management, forest management and nature conservancy. A lack of appropriate river management of the Belá River resulted from our study as well. The key challenge from this point of view is engagement activities in knowledge exchange among stakeholders, researchers and public as well as involve local communities in the decision-making process as far as possible by sharing evidence, listening to the ideas and assessing priorities. The challenge of sustaining Belá River in the face of climate change raised as additional issues to respect all strategic documents where support their implementation lead to greater enforcement of existing regulations. The need for better-integrated planning focusing on economic balance to provide multiple benefits to water users (e.g. recreation, hydropower) is crucial to achieving environmental improvements. The importance of wider landscape management and the value of looking at river landscape as an entire system emphasizes attention to protection purposes. Further operative step within these challenges tends to flood risk management coordination with planning the environmentally friendly or nature-based approaches (e.g. room for the river concept). The importance of the main conflict identification based on revealing the management operating shortages support redirection of future activities in the more appropriate way. Then, our suggested compilation of the main management challenges (Fig. 7) for the future is improving the sustainable development of river systems generally not only for the Belá River as part of the Natura 2000 Network case study. On the other hand, we are realizing that further investigation in these issues and more detailed analyses according to a good examples from practice (e.g. from England^79^) is needed.

Although the actual national document from 2019, the Strategy of the Environmental Policy of the Slovak Republic until 2030, promises more responsible water management and a focus on the revitalization and re-naturalization of watercourses and the adjacent riverine landscapes, in the sense of WFD, the question of how Slovakian river managers will deal with the deadline to achieve good water status by 2027 by implementing the practice is still unanswerable.

## Conclusion

The presented intervention in the braidplain area of the Belá River resulted in an undesirable simplification of the river pattern, loss of geomorphic diversity, loss of channel–floodplain connectivity, and disturbance and restraint of hydromorphological continuity. The increased flow velocity, shear stress and stream power recorded for all studied cross-sections on the Belá might result in accelerated erosion of the riverbed in the near future^39^. Moreover, the destruction of Natura 2000 Network habitats (aquatic and terrestrial) is presupposed as well as interruption of the bio-geomorphological effect of LWD.

The geomorphic recovery^10,45^ of the Belá River with a typical multi-thread pattern as well as the richness and diversity of habitats (geodiversity) after river training is desirable. Natural restoration of the transverse continuity of the main channel with the secondary channels on the floodplain can be expertly estimated in the context of climate change and low *n*-year flood occurrence. Flood magnitude was quasi-constant and never exceeded 10-year frequency from 1958 up to 2018^15,33^. A major flood with a destructive (transformative) geomorphological effect on the river morphology, supporting a return to its reference state, is assumptive for a flood with a ˃ 20-year RI.

Preservation of the natural characteristics of riverbed morphology is closely linked to the conservation of its ecological importance and function, which logically implies that one would not exist without the other. Our research findings confirmed the minimization of flood wave mitigation on the floodplain as well as an increase of the erosive force in the main channel of the monitored river reaches after the management intervention. These fundamental analyses help for future management issues as well as for the more critical decision-making process in vulnerable and rare river systems. Presented analyses in this paper is done on one of the chosen multi-thread rivers in Slovakian Carpathians, however the subject and consequences of river engineering works done on the Belá River are important for all braided river systems (fragile, sensitive and unique) and concerns all examples of river training management interventions worldwide. The subject is so important since every year we are losing more and more braided river systems and all water resources. This problem relates with multiple anthropogenic stressors for the purpose of the potential hazards control tends to bed degradation, channel shift and narrowing, e.g. the lower Waitaki River in New Zealand, the middle Piave River in Italy or the lower Dunajec River in Poland^80^. Another problem relating to braided rivers is a declining trend in aquifer levels and low-land spring flows (losing water into groundwater), e.g. the Wairau Plain aquifer in the New Zealand^81^ what affect mainly drinking water sources.

Possibly, we need to learn again how to live with floods (what we always did), thus, the paper is aimed to scholars, river engineers, river managers and fluvial geomorphologists in whose hands are given decision concerning rivers and water of our planet. Respect for strategic documents at national and European level is desirable as well. Furthermore, by this work we would like to strongly recommend the application of nature-based flood protection during river training, which is generally essential for maintaining positive climate geo-biodiversity feedback processes.
